# Reduction of the secondary structure topological space through direct estimation of the contact energy formed by the secondary structures

**DOI:** 10.1186/1471-2105-10-S1-S40

**Published:** 2009-01-30

**Authors:** Weitao Sun, Jing He

**Affiliations:** 1 Department of Computer Science, New Mexico State University, Las Cruces, 88003, USA; 2 Zhou Pei-Yuan Center for Applied Mathematics, Tsinghua University, Beijing, 100084, PR China

## Abstract

**Background:**

Electron cryomicroscopy is a fast developing technique aiming at the determination of the 3-dimensional structures of large protein complexes. Using this technique, protein density maps can be generated with 6 to 10 Å resolution. At such resolutions, the secondary structure elements such as helices and *β*-strands appear to be skeletons and can be computationally detected. However, it is not known which segment of the protein sequence corresponds to which of the skeletons. The topology in this paper refers to the linear order and the directionality of the secondary structures. For a protein with *N *helices and *M *strands, there are (*N*!2^*N*^)(*M*!2^*M*^) different topologies, each of which maps *N *helix segments and *M *strand segments on the protein sequence to *N *helix and *M *strand skeletons. Since the backbone position is not available in the skeleton, each topology of the skeletons corresponds to additional freedom to position the atoms in the skeletons.

**Results:**

We have developed a method to construct the possible atomic structures for the helix skeletons by sampling the solution space of all the possible topologies of the skeletons. Our method also ranks the possible structures based on the contact energy formed by the secondary structures, rather than the entire chain. If we assume that the backbone atomic positions are known for the skeletons, then the native topology of the secondary structures can be found in the top 30% of the ranked list of all possible topologies for all the 30 proteins tested, and within the top 5% for most of the 30 proteins. Without assuming the backbone location of the skeletons, the possible atomic structures of the skeletons can be constructed using the axis of the skeleton and the sequence segments. The best constructed structure for the skeletons has RMSD to native between 4 and 5 Å for the four tested *α*-proteins. These best constructed structures were ranked the 17^th^, 31^st^, 16^th ^and 5^th ^respectively for the four proteins out of 32066, 391833, 98755 and 192935 possible assignments in the pool.

**Conclusion:**

Our work suggested that the direct estimation of the contact energy formed by the secondary structures is quite effective in reducing the topological space to a small subset that includes a near native structure for the skeletons.

## Background

**Figure 1:**
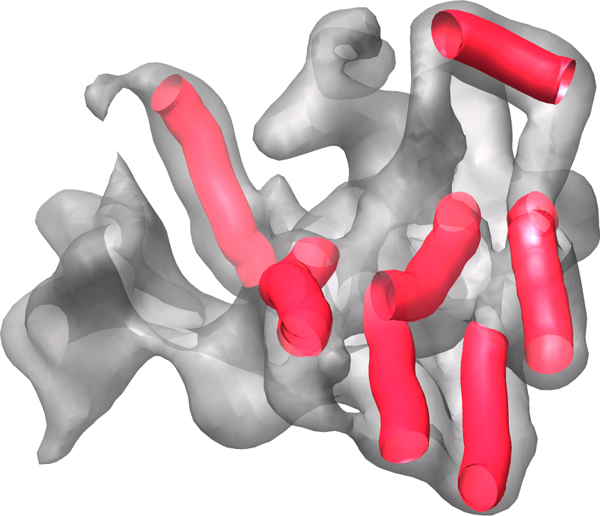
**Protein density map and the detected helix skeletons**. The density map simulated using protein 1AGW (PDB Id) to 10 Å resolution using EMAN [31, 32]. The helix skeletons (sticks) were detected using Helix Tracer [13].

Electron cryomicroscopy (Cryo-EM) is a fast developing experimental technique aiming at the determination of the 3-dimensional structure of large protein complexes [1-11]. Although not yet at the atomic resolution, this technique can be used to generate protein density maps at 6 to 10 Å resolution [1-11]. Computational methods have been developed to detect the secondary structures such as helices and *β*-strands from the density map at this resolution [12-16]. Since the secondary structures are often the major building blocks of a structure, the detected secondary structures appear as the skeletons (marked as sticks in Figure 1) of the density map. In the density map of 6–10 Å resolution, alpha helices look like sausages with 5–6 Å in diameter, and beta-sheets appear as extended volumes with varying sizes. The connection relationship between the skeletons is often unavailable in the density maps.

Given a protein sequence, the location of secondary structures on the sequence can be roughly predicted using existing secondary structure prediction methods. Such methods can generally predict the secondary structures to about 70–80% accuracy [17-19]. Although not completely correct, the predicted secondary structure segments of the sequence provide a rough location of the secondary structures. Wu *et al *has used the secondary structure geometrical relationships existing in the PDB to reduce the topological space. The reduced possible topologies were then used to add the loops and a simulation of the entire chain was used to identify the true topology [20]. We have also shown previously that the decoys generated by Rosetta ab initio structure prediction software can be used to reduce the topological space [21,22]. In this paper, we will introduce a method that directly measures the contact energy of the secondary structures to reduce the topological space of the skeletons. This is the first such demonstration. The secondary structure topology problem of the CryoEM density map is to find the true mapping between the predicted secondary structure segments on the sequence and the skeletons of the density map. Secondary structure topology in this paper refers to the order and the direction of the secondary structures such as helices and strands with respect to the protein sequence. For a protein with *N *helices and *M *strands, there are (*N*!2^*N*^)(*M*!2^*M*^) different topologies. For example, there are *N*! different permutations for assigning *N *helix segments to *N *helix skeletons and two directions to assign a sequence segment to each skeleton.

This paper investigates the following question. Given the skeletons detected in the protein density map, is it possible to derive a small subset of the topological space that contains the native topology of the secondary structures using a direct measurement of the energy formed by the secondary structures? The determination of favourable secondary structure topologies has always been one of the most essential problems in tertiary structure prediction. Although predicting the topology, in general, is as hard as predicting the tertiary structure, it is not clear if it is true when the rough location of the secondary structures is known.

Systematic mutation analysis has shown recently that the native protein sequence for a specific 3-dimensional structure is close to optimal [23]. This is intuitively not surprising since nature might have derived a near optimal sequence to achieve the function through evolution. The research in this paper is guided by the intuition that when the relative geometry of the secondary structures is fixed in the 3-dimensional space, there might only be a small number of topologies that can provide energetically favorable structures. In this paper, we describe a method to perform mutation analysis for the secondary structures. It generates possible atomic structures for the skeletons through the exhaustive search in the topology space of the secondary structures.

## Methods

**Figure 2:**
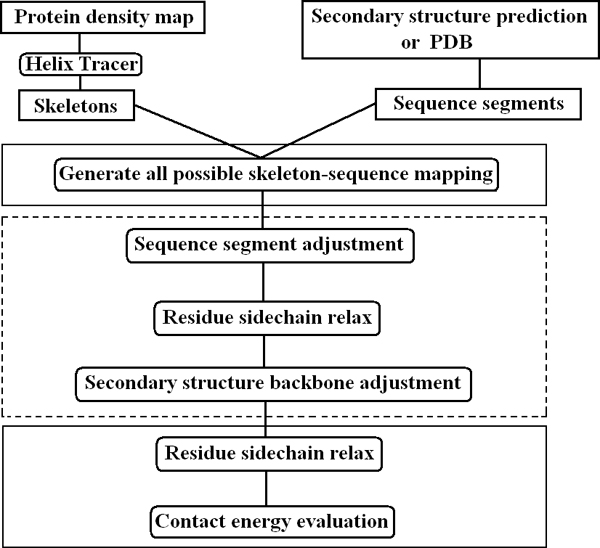
**Generation of the secondary structure topologies for the skeletons**. The three major steps in the first stage are included in two boxes with solid lines. The additional steps in the second stage are marked with a box in dashed lines.

The overall framework to generate the possible secondary structure topologies contains two stages. The first stage is part of the second stage, but it demonstrates the potential of the overall approach. The question for the first stage is how to generate all the topologies if the positions of the C*α *atoms in the secondary structures are given. Note that the C*α *atoms are not resolved in the low resolution density map, although the location of the secondary structures can be computationally detected [12-16]. The second stage starts with the axial location of the skeletons, rather than the backbone atom locations. It generates the coordinates of the C*α *atoms through translation and rotation around the axis. The second stage in this paper only implemented the helices, although the first stage implemented both the helices and *β*-strands. Figure 2 shows a flowchart of the topology generation. We have done a test of stage 1 using 30 proteins. Our preliminary result of stage 2 involves 4 proteins.

### Stage 1 – Secondary structure mutation and side chain optimization

**Figure 3:**
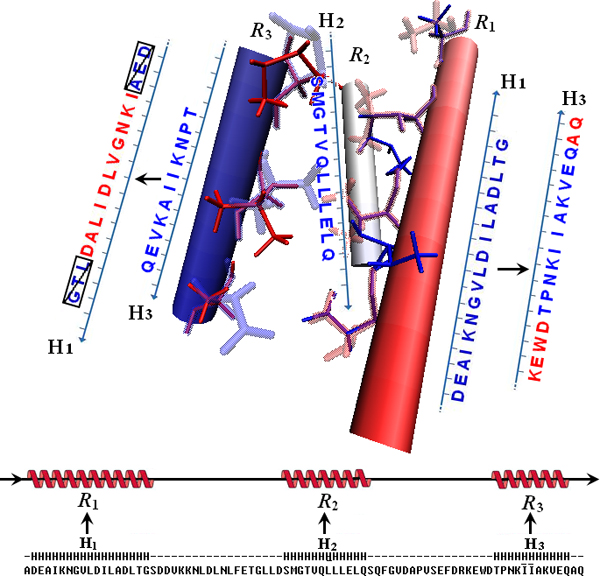
**Secondary structure mutation of **1DV5**(PDB Id)**. The location and orientation of the three helix skeletons (cylinder *R*_1_, *R*_2 _and *R*_3_) are shown. The three segments of the sequence (H1, H2 and H3) forming helices are labelled on the sequence. A mutated topology of the secondary structures was generated by swapping the sequence assignment for (*R*_1_, *R*_3_). Amino acids in the boxes: the deleted amino acids during the mutation; Amino acids in red: the padded amino acid. The thicker side chains are in the native structure. The thinner side chains are in the mutated structure after side chain relaxation. For viewing clarity, only the side chains in the vicinity of the secondary structure interaction are shown.

For each of the tested proteins, a total of 2^*N*^2^*M*^(*N!M!*)of all the possible topologies were generated, where *N *is the number of helices and *M *is the number of beta strands. A mutated topology of the secondary structures was generated using the coordinate of the backbone atoms of the secondary structures and the sequence segments that form the secondary structures. Two major steps were involved: (1) mapping sequence segments to the skeletons and (2) construction of the side chain atoms. Figure 3 shows an example of a native topology (*R*_1 _- *R*_2 _- *R*_3_) and a mutated topology (*R*_3 _- *R*_2 _- *R*_1_) of the secondary structures.

In the native topology, *R*_1_, *R*_2 _and *R*_3 _skeletons were assigned to the sequence segments H_1_, H_2_, and H_3 _respectively. In the mutated topology, *R*_1_, *R*_2 _and *R*_3 _were assigned to H_3_, H_2_, and H_1 _respectively. When the length of the sequence segment (*i.e. *H_1 _in Figure 3) does not match that of the skeleton (i.e. *R*_3 _in Figure 3), we used the length of the skeleton as the reference. The sequence segment was either truncated (crossed boxes in Figure 3) or padded (amino acids in red in Figure 3) based on the distance from the center amino acid of the segment. For example, when *R*_3 _is mutated from H_3 _to H_1_, three amino acids were deleted from each end of H_1 _(Figure 3). When *R*_1 _is mutated from H_1 _to H_3_, H_3 _is padded with six amino acids to match the length of *R*_1_. In this case, since H_3 _is only two amino acids away from the end of the sequence, it is padded more at the other end.

After the sequence segments were assigned to the skeletons, the side chains were constructed. The Rotamer library was used and the side chains were relaxed using the simulated annealing [24,25].

### Multi-well energy function

We have developed a multi-well energy function by incorporating the statistical data to a Lennard Jones shaped function [26]. The multi-well function is composed of the original Lennard Jones shaped function (*U*_0 _in formula (1)) [26] and the statistically derived function (*U*_1 _in (1))using two window functions *f*_1 _and *f*_2_.

(1) $$\begin{array}{l} U(r_{mn})=f_{0}U_{0}(r_{mn})+f_{1}U_{1}(r_{mn})\\ \;=f_{0}\cdot e_{ij}\cdot \left[\frac{q{\left(\frac{r_{0}}{r_{mn}}\right)}^{p}\pm p{\left(\frac{r_{0}}{r_{mn}}\right)}^{q}}{q\pm p}\right]+f_{1}\cdot e_{ij}\cdot \left[\lambda_{r_{mn}}\cdot e^{-\prod_{k=1}^{N_{peak}}{\frac{\left(r_{mn}-r_{k}\right)}{2c^{2}}}^{2}}\right] \end{array}$$

The *e*_*ij *_is the contact energy between two amino acids [27,28]. The *p*, *q *and *r*_0 _are adjustable parameters. *r*_*mn *_is the distance between the side-chain centers of residue *m *and *n*. The signs are chosen to be positive if *e*_*ij *_> 0, or negative if *e*_*ij *_< 0. There is a potential well near the favorable side-chain distance *r*_0 _in *U*_0_. $\lambda_{r_{mn}}$, *N*_*peak *_and *c *are statistically derived parameters based on an analysis of 413 selected proteins from the PDB. Here $\lambda_{r_{mn}}$ is a non-dimensional coefficient varying with distance *r*_*mn *_and represents the depth of the potential well. *r*_*k *_is the position of the *k*-th residue distance peak in the distribution curve of the residue pair distance. Here the window functions *f*_0_, *f*_1 _satisfy:

(2) $$U(r_{mn})=\{\begin{array}{ll} U_{1}(r_{mn}), & r_{1}-\delta \leq r_{mn}\leq r_{N_{peak}}+\delta \\ U_{0}(r_{mn}), & \begin{array}{ccc} r_{mn}<r_{1}-\delta & or & r_{mn}>r_{N_{peak}}+\delta \end{array} \end{array}$$

*r*_1_, $r_{N_{peak}}$ are the first and the last peak positions respectively in the distance distribution. *δ *is a small constant to allow the smooth connection between *U*_1 _and *U*_0 _that are combined using a set of window functions *f*_1 _and *f*_0_. By statistical analysis of 413 proteins in PDB, a new multi-well contact energy function was developed for each of the 210 residue pair interaction. The contact energy measured is the mean of the energy from all the contact pairs in the secondary structures. Both the intra and inter secondary structure energy were considered. The contact energy from a pair of residues within a helix or a strand is the intra energy, and the contact energy from a pair of residues in different helices or stands is the inter energy. The average of inter and intra energy, named as effective contact energy *U*_eff_, was used as a measurement of the stability of the secondary structures. We are preparing another paper to with more details about the construction of the energy function.

### Stage 2 – Sequence assignment to the skeletons

In stage 1, we assumed that the number of detected skeletons and the number of the sequence segments of the secondary structures are the same. In a general case, suppose that there are *Kα *sequence segments forming helices and *Kβ *sequence segments forming *β*-strands in a native protein structure. Let's also suppose that there are *N *helix skeletons and *M *strand skeletons detected from the protein density map. Based on our experience, *Kα *and *Kβ *are usually larger numbers than *N *and *M *respectively. The total number of different sequence combination is:

(3) $$n_{comb}=C_{K\alpha }^{N}C_{K\beta }^{M}=\left(\begin{array}{c} K\alpha \\ N \end{array}\right)\left(\begin{array}{c} K\beta \\ M \end{array}\right)$$

For each set of the sequence segments picked from *Kα *and *Kβ*, the total number of possible sequence assignment is (*N! *× 2^*N*^)(*M! *× 2^*M*^), as was dealt with in the first stage. When not all the secondary structures are detected in the density map, the total number of topologies is:

(4) $$n_{perm}=C_{K\alpha }^{N}C_{K\beta }^{M}\times N!\times 2^{N}\times M!\times 2^{M}$$

When the sequence segments are predicted using a secondary structure prediction tool, such as PSIPRED [29], the predicted segments are often shifted from their corresponding true locations. In order to estimate the magnitude of the computation, let's suppose that each sequence segment can have a shift in both directions with a maximal number of *p *amino acids. Then there are 2*p*+1 possible sequence segments for each true segment. The total number of assignments for the skeletons is

(5) $$n_{total}=C_{K\alpha }^{N}C_{K\beta }^{M}\times N!\times 2^{N}\times {\left(2p+1\right)}^{N}\times M!\times 2^{M}\times {\left(2p+1\right)}^{M}$$

Note that the above estimation of the total assignments assumes each segment having the same maximum number of shifts. In reality, different segments might have different maximum number of shifts depending on the nature of the particular sequence. In the current implementation of stage 2, we allowed the maximum shift to be 1 (*p *= 1) and all the tested proteins are *α*-helical proteins. The total possible assignment number *n*_*total *_is still a huge number, even when *M*, *N*, *p *are relatively small.

In order to ignore the obvious wrong assignment between a sequence segment and the skeleton where their lengths differ significantly, we used a threshold *d *= 6 to require them to have similar lengths.

(6) |*l*_*skl *_- *l*_*seq*_| ≤ *d*

Here *l*_*skl*_, *l*_*seq *_is the length of skeleton and sequence segment.

### Stage 2 – Construction of the atomic structures for the skeletons

Once a set of sequence segments are assigned to a set of helix skeletons, the possible atomic structures can be constructed for the skeletons using the information of the segments and the spatial position of the skeletons. However, since the skeleton does not provide the exact location of the backbone atoms at low resolution, this step generates the possible atomic coordinates of the atoms. By CSAW method [30], the protein structure backbone and the side chains were created and moved to the corresponding spatial positions of the helix skeletons. Each constructed helix structure was then rotated around the central axis of the skeleton as part of the structure adjustment. A rigid shift along the axis was also built in the method, although we did not use it in this paper due to the large solution space. The rotation step size was 30° in this paper. For each candidate structure, side chain Rotamer library was used to relax the side chain overlap and Simulated Annealing method was used to minimize contact energy. Protein structures passed the overlap screening were ranked according to their contact energy formed by the helices.

## Results

The dataset used to test the first stage involves 30 proteins from the Protein Data Bank (PDB). Some of the proteins are listed in the paper of Nanias *et. al *[26]. Others were randomly selected from the proteins that satisfy the following criteria: (1) has single domain (2) has 1.5 Å or better resolution (3) share less than 30% sequence similarity (4) has less than 7 secondary structures, due to the amount of computation. The key question in the first stage was to see if the protein is comfortable when the segments of the sequence are assigned to the skeleton in all possible topologies. Intuitively, when the positions of the backbone atoms are fixed, only a small number of the all possible sequence assignments to the skeletons will be energetically favorable. However, there has not been any data to support this hypothesis and as to how much of the population in the mutated topologies is energetically acceptable. Our program was used to construct the all-atom structure for each topology in the total topological space of (*N! *× 2^*N*^)(*M! *× 2^*M*^). The topologies with collision of atoms after side chain relaxation and those with large variation in the per-pair contact energy were eliminated. The rest of the topologies were ranked based on the contact energy. The numbers of topologies with lower contact energy than that of the native topology is shown in column 7 (Table 1). For example, in the case of protein 1HDJ (row 4), there is only 1 topology (column 7) that has the contact energy lower than that of the native topology, out of total 384 (column 6) topologies (Table 1). It appears that the native topology is ranked within the top 30% among all the topologies. For most of the proteins, there is only less than 5% of the total population with lower energy than that of the native. This finding suggests that if the locations of the backbone atoms of the secondary structures are fixed, only a small portion of the huge topological space will result in energetically stable topologies for the secondary structures.

**Table 1:** The topologies of the secondary structures with lower contact energy than that of the native topology: Stage 1, with the assumption of knowing the backbone C*α *atom positions.

PDB	N_AA_	Pct_N_	N_*α*_	N_b_	N_m_	N_eff_	Pct_eff_
1BW6	56	58.93%	4	0	384	9	2.34%

1DV5	80	46.25%	3	0	48	2	4.17%

1HDJ	77	48.05%	4	0	384	1	0.26%

1K6U	58	44.83%	2	2	64	13	20.31%

1RZL	91	56.04%	4	0	384	4	1.04%

1YRI	35	71.43%	3	0	48	5	10.42%

1FK5	93	53.76%	4	0	384	3	0.78%

1G6X	58	44.83%	2	2	64	4	6.25%

1I2T	61	88.52%	4	0	384	2	0.52%

1WPA	107	85.98%	3	0	48	4	8.33%

2NLS	36	47.22%	1	3	96	27	28.12%

1B0X	72	61.11%	5	0	3840	167	4.35%

1KDX	81	66.67%	5	0	3840	6	0.16%

1NFO	131	83.97%	5	0	3840	2	0.05%

1NKL	78	65.38%	5	0	3840	2	0.05%

1UNK	87	55.17%	5	0	3840	7	0.18%

1A0B	117	78.63%	6	0	46080	9	0.02%

1EIJ	72	68.06%	4	2	3072	27	0.88%

1F1F	88	64.77%	6	0	46080	39	0.08%

1FIO	190	88.95%	6	0	46080	2	0.00%

1ZVA	75	88.00%	2	0	8	0	0.00%

1TQG	105	86.67%	4	0	384	0	0.00%

2CC6	64	71.88%	1	3	96	1	1.04%

2END	137	42.34%	3	0	48	5	10.42%

2G7O	68	80.88%	4	0	384	5	1.30%

1NKD	59	86.44%	2	0	8	0	0.00%

2MHR	118	60.17%	5	0	3840	13	0.34%

1NGR	85	56.47%	6	0	46080	102	0.22%

1USM	77	61.04%	2	4	3072	28	0.91%

2OVG	58	67.24%	3	3	2304	19	0.82%

N_AA_: number of amino acids

Pct_N_: the percentage of amino acids in the secondary structures (helices and strands);

N_*α*_: the number of alpha helix; N_b_: gthe number of beta strands;

N_m_: the gnumber of secondary structure mutations, the cross mutation between a helix and a strand is ignored;

N_eff_: the gnumber of the mutated topologies with lower effective contact energy than that of the native;

Pct_eff_: the percentage of the mutated topologies with lower effective contact energy than that of the native by multi-well function;

We tested the approach in stage 2 with four small *α*-proteins. The helix skeletons were first detected using Helix Tracer [13]. Our program was then used to construct the possible all-atom structures exhaustively. It uses the axis location of the helix skeleton and performs rotational sampling of the C*α *coordinates every 30°. It also allows the inexact positioning of a sequence segment for a maximum of 1 amino acid shift. All the constructed structures of the secondary structures were sorted based on their contact energy. Table 2 shows an example (PDB Id = 1LRE) of the ranked structures for the skeletons. Since the total number of the possible structures is too large, only those with negative contact energy were stored and analyzed. One of the key questions in stage 2 is to see if the native assignment of the sequence segments is still included in the top percentage of the list when only the axes of the skeletons are known. Unlike in stage 1 where the true backbone location was used for the skeleton, stage 2 samples the backbone locations through rotation around the skeleton axis. Therefore, our rotation sampling may not hit the true structure exactly, although a structure close to the native can be reached. In fact, if one applies our approach for a protein with unknown structure, this is the situation encountered. Therefore, it is interesting to see even with 30° of sampling step size, how accurate the resulting structure is. Table 2 illustrates the trend of the constructed structures after they were ranked by the contact energy (column 5). The one with the smallest RMSD to native (column 6 row 5) was identified from the entire list. It appears that the structure with the smallest RMSD to native (4.781 Å), the one most similar to the true structure, was ranked the 17^th ^among all the possible constructed structures for the skeletons. This constructed structure indeed has the true topology with the topology Id = 123000. 123000 (column 3 row 5) means that there are three helices in the skeleton and they were assigned to the correct helix sequence segments with the correct direction. We used the first three digits of the topology label to indicate the permutation of the assignment and the last three digits to indicate the assignment direction for each of skeletons. As an example, a topology label of 213001 means the sequence assignment for the first and the second helix segments is swapped, and the third segment is assigned to the correct skeleton but with an opposite direction from the true direction. The structure that is the most similar to the true structure has a shift of 1 amino acid in the 1st and the 3^rd ^segment respectively (column 3 row 5 of Table 2).

**Table 2:** Constructed atomic structures for the skeletons in 1LRE: The structures are ranked by the contact energy (5^th ^column), and those 32066 structures with negative contact energy are included in the table. The structure with the smallest RMSD to native (4.781 Å) is ranked the 17^th^.

Rank	Topology	Shift	Rot	CE	RMSD
1	123100	[-1, 1, 1]	[1.57, 1.57. 1.57]	-2.066887	7.224

2	123100	[-1, 1, -1]	[1.57, 1.57. 3.66]	-2.066817	7.517

...	...	...	...	...	...

17	123000	[1, 0, 1]	[5.76, 3.14, 1.57]	-2.004894	4.781

...	...	...	...	...	...

32066	123011	[1, -1, -1]	[1.05, 1.05, 0]	1.09E-4	12.979

Rank: the rank of the structure by the contact energy (5^th ^column)

Topology: the topology Id, the 1^st ^half of the digits: permutation of the assignment, the last half of the digits: directions (0 or 1) of the assignment for each helix;

Shift: the amino acid position shift from the true helix segment for each assignment, "-" left, "+" right;

Rot: rotation angle around the skeleton axis for each helix, in radian;

CE: Effective contact energy of the constructed helices;

RMSD: the root mean square deviation of the C*α *atoms between the constructed candidate structure and the native structure, in Å.

The results of the four tested proteins are summarized in Table 3. For each of the proteins, a table similar to Table 2 was constructed (data not shown). Then the structure with the smallest RMSD to native from each of the four tables is included as a row in Table 3. For all the four proteins, the structure most similar to the native was ranked in the top portion of the huge list. For example, in the case of 1GXGA, such a structure was ranked the 5^th ^based on its contact energy comparing to other assignments. In this case, Helix Tracer only detected three of the four helices in the true structure. Therefore, only three helices (1, 2, and 4) were used in the calculation. Our direct estimation of the contact energy formed by the secondary structures appears to be quite effective for the four proteins. The structure that is the most similar to the native structure was ranked at the 17^th^, 31^st^, 16^th^, and 5^th ^out of 32066, 391833, 98755 and 192935 constructed structures for the four tested proteins respectively (column 2 and 3 of Table 3). The RMSD to native of the best constructed structure is between 4 Å and 5 Å for all the four proteins. The small RMSD to native value suggests that the best constructed structure is close to the native structure for all the four proteins.

**Table 3:** The constructed structure with the smallest RMSD to native for the four tested proteins.

Protein	Assignments	Rank	Topology	Shift	Rot	CE	RMSD
1LRE	32066	17	123000	[1, 0, 1]	[5.76, 3.14, 1.57]	-2.004894	4.781

1JW2A	391833	31	12340000	[-1, -1, 1, 0]	[0, 0, 3.66, 5.76]	-1.751024	4.718

1DP3A	98755	16	123000	[-1, 0, -1]	[4.71, 5.23, 1.05]	-2.414033	4.341

1GXGA	192935	5	124000	[-1, 0, 1]	[5.23, 3.66, 2.09]	-2.784552	4.665

Protein: the PDB Id;

Assignments: the total number of assignments with the negative contact energy

Rank: the rank of the structure with the smallest RMSD to native;

Topology: the topology Id, the 1^st ^half of the digits: permutation of the assignment, the last half of the digits: directions (0 or 1) of the assignment for each helix;

Shift: the amino acid position shift from the true helix segment for each assignment, "-" left, "+" right;

Rot: rotation angle around the skeleton axis for each helix, in radian;

CE: Effective contact energy of the constructed helices;

RMSD: the root mean square deviation of the C*α *atoms between the constructed candidate structure and the native structure, in Å.

**Figure 4:**
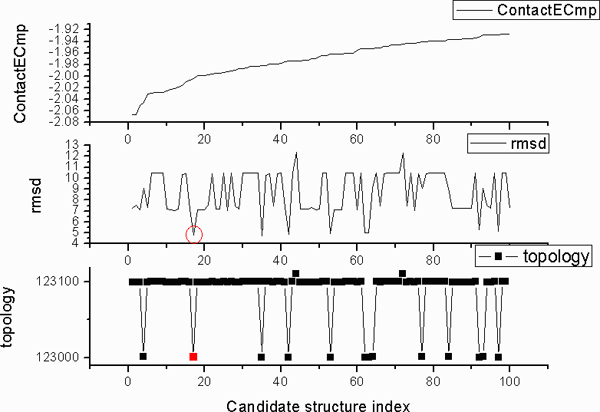
**Top 100 structures for the skeletons in **1LRE**(PDB Id)**. The constructed atomic structures for the helix skeletons were ranked by the contact energy (top panel). A topology Id (bottom panel) includes six digits. The first three digits represent the permutation of the assignment, and the last three represent the relative direction (0, or 1) between the sequence segment and the skeleton for each of the skeletons. The RMSD (middle panel) to the native structure is shown for each of the 100 constructed structures for the skeletons. The constructed structure with the smallest RMSD to native (the 17^th ^of the 100) is marked in red for its topology and the RMSD.

To illustrate the nature of the top ranked structures, we show a distribution of the topologies, the contact energy, and the RMSD to native for the top 100 constructed structures in Figure 4. The structures of the skeleton of 1LRE were ranked by the contact energy of the secondary structures (top penal of Figure 4). The two most popular topologies in the top 100 structures are 123100 and 123000, although a few 123110 (*i.e. *rank between 70 ~80) and 123001 (*i.e. *rank between 60~70) exist (lower panel of Figure 4). Here, the topology Id of 123000 means the three helix segments of sequence were correctly assigned to the corresponding skeletons with the correct directions. A topology ID of 123100 means the three helix segments of the sequence were correctly assigned to the skeletons. However, the direction of H_1 _sequence segment was assigned to the opposite direction from the true direction. Note that a wrong topology (*i.e. *123100) can be energetically favourable, since we observed that the structure with the lowest contact energy has a wrong topology. However, our result shows that the great majority of the wrong topologies correspond to energetically non-favorable structures. The near native structure of the skeletons can be found near the top of the list, the 17^th ^that are marked in red (middle and lower panel of Figure 4).

## Discussion

We introduced a method to construct the possible atomic structures for skeletons of the density map. The method is built on the direct estimation of the contact energy formed by the skeletons. The evaluation of the constructed structures is independent from the knowledge of the conformation of the entire protein chain. This approach can be potentially useful when the size of the protein is large where the ab initio prediction of the entire chain is often hard. Our approach suggests that it is possible to derive a set of possible topologies of the secondary structures without constructing the conformation of the entire chain. Although our approach is expected to encounter the huge computation expenses when large proteins are the targets, it is interesting to see if the approach can be optimized to tackle the computation problem.

We performed a small test in stage 2 using four *α*-proteins. Our current method samples the possible structures for the skeletons using a fixed step size. The advantage of this sampling method is that it provides details about the solution space. The disadvantage obviously is its computational expense. This approach was able to rank the most near native structure at the 17^th ^of 32066, the 16^th ^of 98755, the 5^th ^of 192935 and the 31^st ^of 391833 structures for 1LRE, 1DP3A, 1GXGA, and 1JW2A respectively (Table 3). Note that the base number here only includes the structures with negative contact energy, and not the entire topological space. A test including more proteins, particularly the non-alpha proteins, will provide more concrete results. It is also possible to use simulated annealing in stead of the exhaustive sampling of the topological space.

We have developed a method to eliminate the secondary structure topologies through an direct estimation of the contact energy of the secondary structures. It will be interesting to see how stable this screen method is compared to a geometrical screening method introduced by Wu *et. al *[20]. It is possible that the combination of the two approaches can be more effective in reducing the topological space for the skeletons.

## Conclusion

This paper explores the question if it is possible to derive a small set of atomic structures for the skeletons through the sampling of the entire topological space of the secondary structures. We have developed an approach to enumerate the possible atomic structures for the skeletons and to rank the structures using a direct estimation of the contact energy formed by the secondary structures. We demonstrate for the first time that it is possible to use the secondary structure contact energy to eliminate the great majority of the wrong structures for the skeletons. A test of 30 proteins suggests that when the backbone atoms are given for the skeletons, there are only a small number of topologies with more comfortable structures than that of the native. Even when the backbone atoms are not known for the skeletons, it is still possible to construct a small set of structures that contains a near native structure for the skeletons. Our results suggest that without constructing the structure of the entire chain, it is still possible to derive a small set of the atomic structures for the skeletons, out of the huge solution space, using our recent development of the multi-well energy function.

## Competing interests

The authors declare that they have no competing interests.

## Authors' contributions

WS and JH developed the methodology. WS developed the code and performed the tests. JH provided overall guidance. Both are involved in developing the manuscript.
